# A theoretical study of new polar and magnetic double perovskites for photovoltaic applications

**DOI:** 10.1039/d2ra06478b

**Published:** 2022-11-30

**Authors:** Neda Rahmani, Alireza Shabani, Jost Adam

**Affiliations:** Niels Bohr International Academy, Niels Bohr Institute, University of Copenhagen Jagtvej 160 København Ø 2100 Denmark neda.rahmani@nbi.ku.dk; Department of Electrical and Photonics Engineering, Photonic Nanotechnology, NanoPhoton – Center for Nanophotonics, DTU Ørsteds Plads, 345A, 276 Kgs. Lyngby 2800 Denmark; Computational Materials Group, SDU Centre for Photonics Engineering, University of Southern Denmark, Campusvej 55 Odense 5230 Denmark jostadam@sdu.dk

## Abstract

Searching for novel functional materials has attracted significant interest for the breakthrough in photovoltaics to tackle the prevalent energy crisis. Through density functional theory calculations, we evaluate the structural, electronic, magnetic, and optical properties of new double perovskites Sn_2_MnTaO_6_ and Sn_2_FeTaO_6_ for potential photovoltaic applications. Our structural optimizations reveal a non-centrosymmetric distorted triclinic structure for the compounds. Using total energy calculations, antiferromagnetic and ferromagnetic orderings are predicted as the magnetic ground states for Sn_2_MnTaO_6_ and Sn_2_FeTaO_6_, respectively. The empty d orbitals of Ta^5+^-3d^0^ and partially filled d orbitals of Mn/Fe are the origins of ferroelectricity and magnetism in these double perovskites resulting in the potential multiferroicity. The studied double perovskites have semiconducting nature and possess narrow band gaps of approximately 1 eV. The absorption coefficient (*α*) calculations showed that the value of *α* in the visible region is in the order of 10^5^ cm^−1^. The structural stability, suitable band gap, and high absorption coefficient values of proposed compounds suggest they could be good candidates for photovoltaic applications.

## Abstract 2

Herein, the authors theoretically investigate the structural, electronic, magnetic, and optical properties of new double perovskites Sn_2_{Mn,FE}TaO_6_. Their stability, band gaps, and high absorption coefficients suggest them for future photovoltaics.

## Graphical abstract

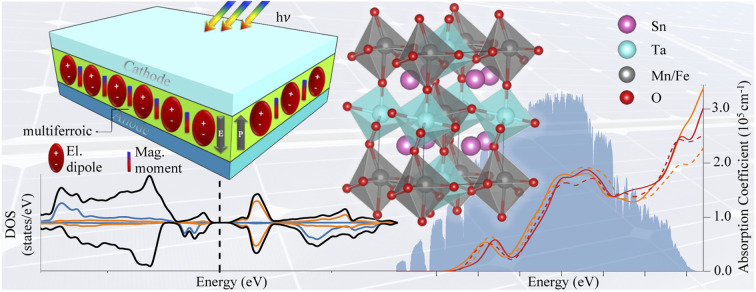

## 1 Introduction

Our society constantly needs technological innovations for a more sustainable future. Due to the continuous increase of energy demand, creating clean energy resources and designing multifunctional materials have become a global issue. Optimal use of clean solar energy as a renewable and abundant source through photovoltaic (PV) conversion of solar photons into electrical energy is an environmentally friendly solution to the current energy crisis. The discovery of the bulk PV effect in ferroelectric (FE) perovskites, which is quite different from that of the traditional p–n junction PVs, has paved the way for exploring new approaches for energy conversion.^1,2^ In conventional p–n junction-based PV devices, the photo-generated charge carriers are driven by the electric field created at the depletion region. Whereas, in FE materials without the need for p–n junction, electric polarization induces a strong internal electric field, which can be used to separate photo-generated charge carriers, thus producing a photovoltage. FEs are typically wide band gap materials (∼3–4 eV), making them unsuitable for PV applications. Therefore, reducing the band gap without affecting the FE properties is necessary to obtain and develop competitive PV devices. Most recently, multiferroic (MF) materials with the coexistence of ferroelectricity and magnetism in the same phase show promise as candidates for harvesting energy, especially from sunlight. Due to the influence of magnetic ordering on electron–electron interaction, MFs have relatively smaller band gaps which is one of the key parameters to harness electrical power from sunlight, as well as improved photocurrents compared to conventional FEs, making them applicable as active elements in PV devices.^3^ MF systems have recently been explored as alternative materials for PV applications among the next generation of solar cell technologies.^4–6^ For instance, BiFeO_3_, as one of the most studied MF systems with a band gap of ∼2.6–2.7 eV, has been intensively investigated in view of its use in PV devices.^7–9^ Nevertheless, a lower band gap facilitates the improved PV performance of such compounds as recently Bi_2_FeCrO_6_ with a tuneable band gap in the range of ∼1.4–2.1 eV is highly promising for PV applications.^4,10,11^ These results draw an intense theoretical research interest to look over a broad range of possible MFs for solar cells. On the other hand, to solve the metal toxicity and instability issues of conventional perovskite solar cells, lead-free double perovskites (DPs) are a noteworthy choice because of their favourable PV properties, intrinsic chemical stability, and environmental friendliness. The lead-based MFs such as PZT, PLZT, and PbTiO_3_ are extensively studied.^12–14^ Nevertheless, due to health and environmental concerns, the practical applications of these materials are also limited. Oxide materials, including perovskites and DPs, can be cost-friendly and widely available. Their properties like electrical energy, ferromagnetic (FM) and FE effects, and bandgap energy are in a favorable zone. Given that MF materials for PV applications are still not thoroughly investigated, it is strategically important to focus on designing these materials from an electronic point of view to engineering their band gap as a powerful technique for designing new materials and devices. In this work, we theoretically explored the structural, electronic, magnetic, and optical properties of new DPs Sn_2_MnTaO_6_ (SMTO) and Sn_2_FeTaO_6_ (SFTO), based on density functional theory (DFT). In search of multiferroics, we have designed polar and magnetic double perovskites in which the polarization can be attributed to the second-order Jahn–Teller (SOJT) effect of Ta^5+^: d^0^ ions and magnetism is due to the 3d transition metals Mn and Fe. Our calculations show that SMTO and SFTO possess significant structural distortion and semiconducting behaviour with band gaps within the optimal range of PV devices. To the best of our knowledge, there is no experimental work, and this is the first theoretical prediction for SMTO and SFTO compounds, so we hope this research serves as a reference and guidance for applying these new materials for PV applications.

## 2 Numerical methods

We performed the first-principles simulations using the SIESTA code^15^ in which the wave functions of valence electrons, that is, the solutions to the Kohn–Sham Hamiltonian eigenvalue problem, were expanded by double zeta polarization (DZP) basis set.^16^ SIESTA uses a linear combination of atomic orbitals (LCAO) with localized basis set from spherical functions. The generalized gradient approximation (GGA) in Perdew–Burke–Ernzerhof (PBE) form^17^ was employed to approximate the exchange–correlation functional parameters. Following the convergence test, we sampled the Brillouin zone (BZ) by 7 × 7 × 5 Monkhorst–Pack *k* points,^18^ and chose the cut-off energy 300 Ry. For the geometry optimization, the maximum atomic force was set to 0.01 eV Å^−1^ and 1 × 10^−4^ eV energy difference for the convergence criteria.^19–21^ To investigate the electronic and magnetic properties of SMTO and SFTO DPs, including transition metals with localized d orbitals that need strong correlation electron systems, GGA + *U* calculations were carried out, which is a better description than GGA.^22^ Here *U* was set to 4.0 eV and 5.0 eV for the 3d ions Mn and Fe, while the 5d Ta ions were weakly correlated with *U* = 1.0 and 2.0 eV, which are reasonable for 3d and 5d ions according to the literature.^23–25^ To obtain the optical response, one needs to calculate the optical matrix element showing the transition probability of electrons from a lower-energy state to a higher state upon the absorption of an electromagnetic wave. This is possible by sandwiching the momentum operator of an electron between two Kohn–Sham electronic states and taking into account the energy conservation considerations and filled and empty states probabilities for electrons.1
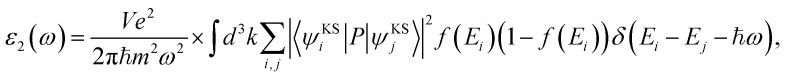
where *ψ*^KS^_{*i*,*j*}_ are the Kohn–Sham Hamiltonian's eigenstates (wave functions), *ℏω* is the incident photon energy, *P* is the electron momentum operator, *f* is the Fermi distribution, *e* is the electron's electric charge, *V* is the unit cell volume, *m* is the electron mass, and *i* and *j* denote filled initial and empty final states, respectively. Furthermore, *δ* indicates Dirac's delta distribution, *ω* is the (angular) irradiation frequency, and *E*_*i*_ and *E*_*j*_ denote the energy of initial and final states. The function *ε*_2_ thereby defines the material's optical absorption behavior, and one defines the overall, frequency-dependent, optical response function by

2 *ε*(*ω*) = *ε*_1_(*ω*) + i*ε*_2_(*ω*),

where the real part *ε*_1_ relates to the imaginary part *ε*_2_*via* the Kramers–Kronig relationship,^26–28^ and it describes the scattering power of a material. The absorption coefficient can be obtained from the real and imaginary parts of the dielectric function as *α*(*ω*) = 2*ωk*(*ω*)/*c*, and *k* is the refractive index’ imaginary part, which relates to the relative permittivity *ε* as 
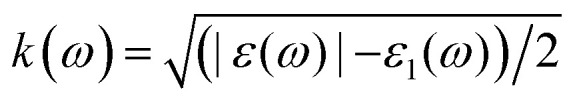
 (ref. 29). The optical properties, including dielectric functions and absorption spectrum, were computed using a denser *k* mesh of 30 × 30 × 30 elements.

## 3 Results and discussion

### 3.1 Geometry optimization and stability of perovskite structures

We started our calculations by finding the ground state structures of SMTO and SFTO DPs. In that light, we considered three different crystal structures of cubic (space group *Pm*3̄*m*, *a* = 8.09), tetragonal (space group *I*4/*m*; *a* = *b* = 5.50, *c* = 8.00), and monoclinic (space group *P*21/*n*; *a* = 5.43, *b* = 5.74, *c* = 7.90; 85.59, 90.00, and 90.00), leaning on literature reports for similar double perovskites.^30–32^ After performing structural optimizations, including atomic positions and lattice parameters, we found that the considered monoclinic structure yields lower total energy than the tetragonal and cubic structures. The calculated structural parameters listed in Table 1 indicate that after optimization, the symmetry is further decreased to triclinic symmetry. Therefore, the triclinic (space group *P*1) was determined to be the ground-state crystal structure of both compounds.

**Table 1:** Calculated ground-state structural parameters of Sn_2_MnTaO_6_ and Sn_2_FeTaO_6_ DPs, namely tolerance factor (*t*), octahedral factor (*μ*), lattice parameters, and cell volumes

Space group	Sn_2_MnTaO_6_	Sn_2_FeTaO_6_
	Triclinic (*P*1)	Triclinic (*P*1)
*t*	0.89	0.89
*μ*	0.46	0.46
*a* (Å)	5.63	5.60
*b* (Å)	5.88	5.82
*c* (Å)	8.36	8.29
*α* (°)	88.18	88.49
*β* (°)	89.03	89.29
*γ* (°)	92.02	91.21
*V* (Å^3^/f.u.)	276.82	270.09

**Fig. 1:**
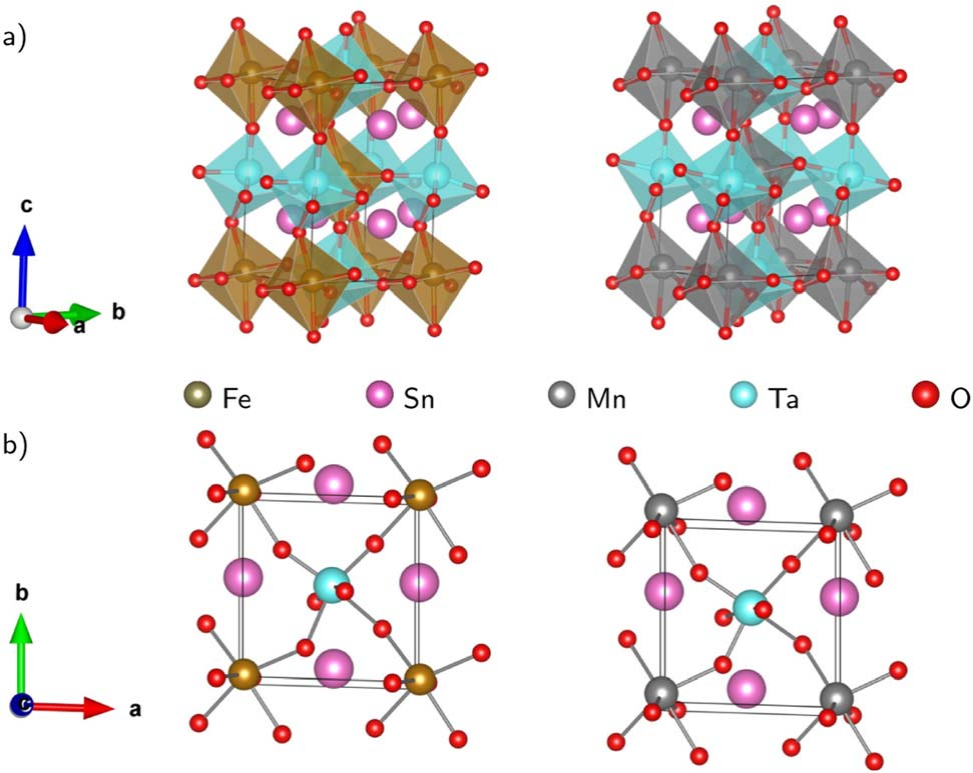
Schematic representation of the Sn_2_MnTaO_6_ and Sn_2_FeTaO_6_ double perovskites conventional unit cell in (a) polyhedral form and (b) top view of the atomic form. The deviation of B–O–B′ bond angles from 180° indicating significant buckling of the octahedral connection. The Jahn–Teller effect is responsible for the BO_6_ and B′O_6_ octahedra distortion resulting in B/B′–O bond length changes (see 〈*ω*〉 and Δ*d* in Table 2).

Fig. 1(a) displays the relaxed conventional unit-cell of SMTO and SFTO containing 20 atoms having two formula units. For application analysis, the studied materials must be structurally and thermodynamically stable. To assess the geometrical stability and perovskite formability of A_2_BB′O_6_ system, we calculated the octahedral factor *μ* and the Goldschmidt tolerance factor *t* presented in eqn (3) (ref. 33). The octahedral factor *μ* is the ratio between the ionic radius of B-site cation (*r*_B_) and that of oxygen ion (*r*_O_), predicting the stability of the BO_6_ octahedra. The tolerance factor *t* applies the ionic radii of A, B, B′ and O ions (*r*_A_, *r*_B_, *r*_B′_, and *r*_O_) to estimate the tolerable size mismatch of ions and predict the formability of the stable perovskite crystal.3
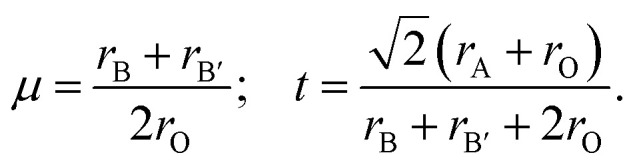


Calculating the octahedral factor *μ* and tolerance factor *t*, derived from simple geometrical principles, is an easy way to predict the basic formability of any new material with targeting perovskite structure based on chemical composition. The conditions proposed for these two parameters suggesting a stable perovskite structure are 0.410 < *μ* < 0.895 and 0.813 < *t* < 1.107.^34–36^

The tolerance factor *t* value gives an initial idea of the crystal structure type. The ideal cubic symmetry (space group *Pm*3̄*m*) with B–O–B′ bonding angles of 180° is usually predictable when the value of *t* is close to unity (1.0 < *t* < 1.05). The deviation of *t* from 1.0 causes different crystal structures as for *t* > 1.05, a hexagonal symmetry (space group *R*3̄*m*) is expected. If 0.97 < *t* < 1.0, the DP compounds have a tetragonal symmetry (space group *I*4/*m*), whereas with a decreasing tolerance factor *t* < 0.97, the materials tend to crystallize into distorted perovskite structures such as an orthorhombic symmetry (space group *Pnma* or *Pbnm*) or a monoclinic symmetry (*P*21/*n*).^25,37^ The deviation from the cubic symmetry occurs due to the octahedral distortion, which is often accompanied by tension and compression in the three bonds A–O, B–O, and B′–O due to the mismatch compensated by tilting and rotation of BO_6_ and B′O_6_ octahedra. These distortions occur to obtain a more favourable configuration from the energetic point of view.

Using available ionic radii,^38^ our calculations gave the value of 0.46 and 0.89 for *μ* and *t*, respectively for SMTO and SFTO, which are within the recommended range for stable perovskite structures. Such a value for *t* is generally observed for significantly distorted perovskites.^39^ Since the Mn^3+^ and Fe^3+^ ions have identical ionic radii (0.645 Å), for the same cations of Sn and Ta at A and B′ sites, respectively, SMTO and SFTO DP have the same value of *μ* and *t*.

The calculated *t* values suggest distorted structures for our investigated compounds. The regularity of the octahedra distortion can be quantified using the octahedral bond-length distortion parameter that can be obtained as4
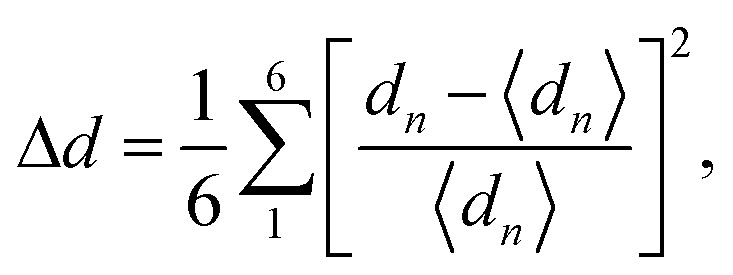
where the individual B(B′)–O bond distances are given by *d*_*n*_ and 〈*d*_*n*_〉 is the average of B(B′)–O bond lengths.^40^ The obtained distortion parameter values for Mn/FeO_6_ and TaO_6_ octahedra in SMTO and SFTO are listed in Table 2. The results show that MnO_6_ octahedra undergo a higher distortion than FeO_6_, which can be attributed to the Jahn–Teller (JT) effect due to the electron configuration of Mn^3+^: 3d^4^. The JT theorem predicts that removing the two- and three-fold electronic degeneracy of high-energy e_g_(d_*z*^2^_,d_*x*^2^−*y*^2^_) and low-energy t_2g_(d_*xy*_, d_*xz*_, d_*yz*_) orbitals, respectively, provide an energetic stabilization geometry resulting in an octahedral distortion.^41^ The value of Δ*d* for significantly distorted octahedra is in the range of 3.3 × 10^−3^ to 5.0 × 10^−3^ (ref. 42). Thus, as shown in Table 2, the calculated TaO_6_ octahedral distortion in SMTO and SFTO compounds are comparatively high due to the non-centrosymmetric displacement of Ta^5+^ (3d^0^) atoms in their respective octahedra (SOJT effect). This off-centre shift of the Ta atom within TaO_6_ octahedral may form a permanent dipole moment in the crystal and provide the main deriving force for ferroelectricity. The ferroelectricity originated from SOJT effect has been reported for some Ta-based perovskites such as Zn_2_FeTaO_6_ (ref. 39). These results coupled with the considerable deviation of the B–O–B′ bond angle form 180° as can be described by distortion angle 〈*ω*〉 = 180 − 〈*θ*〉, in which 〈*θ*〉 is the average B–O–B′ bond angle in B(B′)O_6_ octahedral. The relatively high value of 〈*ω*〉 can be explained by the fact that, in both studied cases, the A-site cation is too small to remain in contact with the anions. Therefore, the B–O–B′ links bend slightly, and the B(B′)O_6_ octahedra tilt to bring some anions into contact with the A cations. Tilting the B(B′)O_6_ octahedra change the overlap between the transition metals B(B′) d states and O-2p states having a significant impact on the perovskite properties.

**Table 2:** Calculated geometrical parameters using GGA

Parameters	Sn_2_MnTaO_6_	Sn_2_FeTaO_6_
〈Ta–O〉	2.07	2.09
〈Mn/Fe–O〉	2.28	2.17
〈*ω*〉 (Mn/FeO_6_)	33.99	35.35
〈*ω*〉 (TaO_6_)	34.20	35.56
Δ*d* (TaO_6_) × 10^−3^	2.91	5.23
Δ*d* (Mn/FeO_6_) × 10^−3^	1.43	1.37
*E*_form_	−3.35	−7.62

To understand the thermodynamic stability of the studied DP systems, we calculated the formation energy *E*_form_ by calculating the energy difference of the DP structure and existing materials using the expression

5 *E*_form_(Sn_2_BTaO_6_) = *E*_tot_(Sn_2_BTaO_6_) − (2*E*_Sn_ + *E*_B_ + *E*_Ta_ + 6*E*_O_).

Our calculations yield *E*_form_ values of −3.35 and −7.62 eV for SMTO and SFTO, respectively. They are negative values, indicating potential stable structures and favoring the formation of the studied compounds.^43–45^

### 3.2 Magnetic properties

After ensuring the ground state crystal structure stability, we calculated the magnetic and electronic properties of the predicted compounds using the optimized structural information. As mentioned before, to capture the correct magnetic and electronic properties, it is crucial to include the electron–electron correction beyond GGA at Mn/Fe (3d) and Ta (5d) sites by applying Hubbard correction. We utilized spin-polarized DFT calculations with the GGA + *U* techniques to assess the magnetic and electronic properties of the chosen compounds. Here, we tested values of *U* = 4, 5 eV for Mn/Fe-3d and *U* = 1, 2 eV for Ta-5d. We note that the magnetic and electronic behavior do not strongly depend on the choice of *U* values, as there is a convergence for total magnetic moments and band gaps with respect to the *U* values. Therefore, for the sake of simplicity, we only show the results of *U*_41_ (*U*_Mn,Fe_ = 4 eV and *U*_Ta_ = 1 eV).

**Fig. 2:**
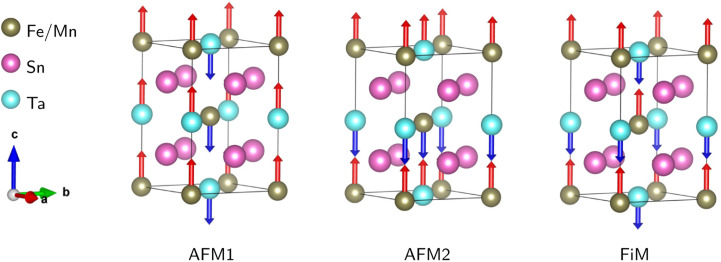
Different magnetic configurations of the conventional cell for the Sn_2_Mn/FeTaO_6_ DPs; AFM1, 2: antiferromagnetic; FiM: ferrimagnetic. Total energy calculations show that AFM2 and FM orderings are the magnetic ground states for SMTO and SFTO, respectively, resulting from a superexchange interaction between Mn/Fe-3d half-field and Ta-5d empty orbitals following the Goodenough–Kanamori rule.

To confirm the stable magnetic state of SMTO and SFTO DPs in their triclinic crystal phase, total energy calculation was performed in which we assume different magnetic ordering of FM, antiferromagnetic (AFM1, 2), and ferrimagnetic (FiM) by the spin alignments of the Mn/Fe and Ta ions Fig. 2. The total energy calculations show the stability of AFM2 state over FM state by a relatively small energy difference of 0.20 meV for SMTO, while the FM phase has the minimum energy for SFTO. Table 3 summarizes the calculated total energy differences between FM and AFM orderings (Δ*E* = *E*_FM_ − *E*_AFM_), total and local magnetic moments for the investigated compounds. The Mn and Fe atoms dominate the magnetic behaviour, with magnetic moments of 5.03 *μ*_B_ and 3.98 *μ*_B_ in SMTO and SFTO, respectively. The zero-values for the total magnetic moments and local magnetic moments with different signs confirm that the Mn ions order antiferromagnetically with respect to the Ta ions in SMTO. The Mn_1_ spin moment is completely compensated by the Mn_2_ spin moment. In contrast, the magnetic moments of Fe and Ta are parallel in SFTO. From the formability point of view, the charge balance between cations and anions must be maintained to form a perovskite structure. The calculated magnetic moments at Mn/Fe and Ta sites indicate the stabilization of nominal +3 and +5 states with d^4^/d^5^ and d^0^ occupancies, respectively, keeping the charge neutrality in the ionic picture of Sn^2+^_2_ (Mn/Fe)^3+^Ta^5+^O_6_^2−^. It puts the transition metals in the electron configuration of Mn^3+^(3d^4^: t^3^_2g_ e^1^_g_), Fe^3+^(3d^5^: t^3^_2g_ e^2^_g_), and Ta^5+^: 5d^0^, resulting in an AFM superexchange between Mn-3d half-field states and a FM superexchange between Mn/Fe-3d half-field and Ta-5d empty orbitals following the Goodenough–Kanamori rule.^46,47^ The d-states of B (B′)-site cations split into low-energy t_2g_ triplet and high-energy e_g_ doublet subsets driven by the crystal field in the octahedral environment. In the +5-oxidation state, Ta-d states remain empty, and the small value of magnetic moment comes from the strong hybridization of Ta-5d with O-2p states.

**Table 3:** Calculated total energy difference (Δ*E*), magnetic moments, and band gaps of SMTO and SFTO by GGA + *U*

Material	Δ*E*_FM−AFM_ (eV)	Total magnetic moment (*μ*_B_)	Local magnetic moments (*μ*_B_)	Band gap (eV)	
				↑	↓
Sn_2_MnTaO_6_	0.0002	0.00	*m*_Mn1_ = 5.04, *m*_Ta1_ = −0.03	1.02	1.17
			*m*_Mn2_ = −5.02, *m*_Ta2_ = 0.04		
Sn_2_FeTaO_6_	−0.02	8.00	*m*_Fe1_ = 3.99, *m*_Ta1_ = 0.06	1.08	1.06
			*m*_Fe2_ = 4.00, *m*_Ta2_ = 0.07		

### 3.3 Electronic properties

**Fig. 3:**
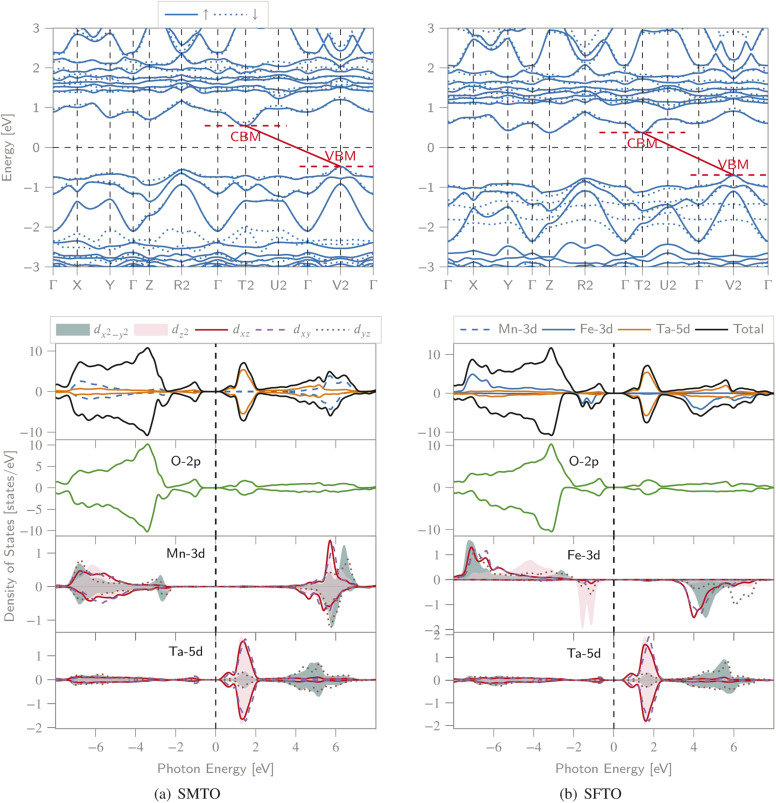
The spin polarized band structure (upper), total DOS and PDOS (lower) of the (a) SMTO and (b) SFTO compounds computed employing GGA + *U*, *U*_41_ approximation (*U*_Mn,Fe_ = 4.0 eV and *U*_Ta_ = 1.0 eV). The Fermi level is set as zero. CBM: conduction band minimum, VBM: valence band maximum. Both structures exhibit a semiconducting nature with band gaps slightly larger than 1.0 eV in both spin states, see Table 3 for details.

We calculated the spin-polarized band structures along the high symmetry direction of BZ, total and partial DOS (PDOS) (Fig. 3). Both spin channels comprise band gaps, resulting in a semiconducting nature for both compounds. For SMTO, there are indirect band gaps for the spin-up and spin-down channels, of 1.02 eV and 1.17 eV, respectively, as the valence band maxima (VBM) and conduction band minima (CBM) are located at the V_2_ and T_2_ symmetry points of BZ, respectively. SFTO has the same behavior with indirect band gaps of 1.08 eV and 1.06 eV for the spin-up and spin-down channels, respectively (along the V_2_ → T_2_ path).

For SMTO, we find that the most contribution in the valence band from −7.0 to −2.0 eV below the Fermi level comes from Mn-3d and O-2p states, while the Ta ion does not contribute significantly to PDOS in this region. Above the Fermi level, in the energy region between 0.0 and 2.0 eV, the Ta-5d states have the most contribution in the conduction band. In this energy region, the hybridization between O-2p and Ta-5d orbitals causes the non-centrosymmetric displacement of Ta^5+^ ions in their respective octahedra. Below the Fermi level, Ta-5d states are relatively empty, confirming the reported Ta^5+^: 5d^0^ electron configuration. An overlap between the Mn-3d and O-2p states extending from about −7.0 eV to −1.0 eV indicates the Mn–O covalent bonding. A similar behavior is observed for SFTO as in the range of −8.0 eV to −1.0 eV the valence band is mainly composed of O-2p and Fe-d orbitals. The Ta states contribute most significantly to the DOS near the Fermi level in the conduction band, between 0.0 and 2.0 eV.

Moreover, the symmetric DOS distribution for the spin-up and spin-down channels supports the AFM configuration for SMTO. For both SMTO and SFTO, in the range from −7.0 eV to −1.0 eV below the Fermi level and from 1.0 eV to 7.0 eV above the Fermi level, we observe an energy degeneracy for the p and d orbitals of oxygen and Mn(Fe)/Ta, respectively.

### 3.4 Electron charge density analysis

**Fig. 4:**
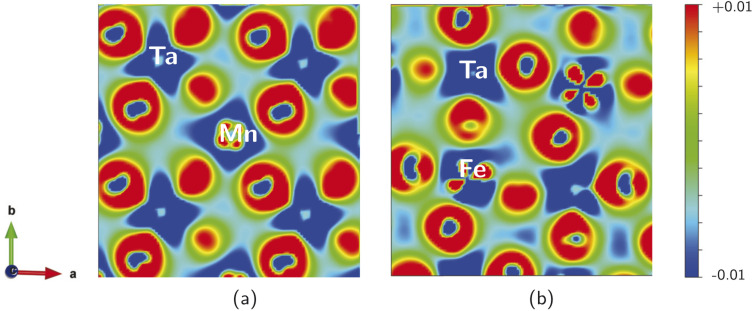
Charge density difference plots (*e*/Å^3^), describing the charge distribution difference between the optimized and initial structures, in (001) plane for (a) SMTO and (b) SFTO compounds. Positive (negative) values show the charge accumulated (depleted) regions. The charge sharing between the Mn/Fe–O and Ta–O atoms in Mn/FeO_6_ and TO_6_ octahedra show the covalent nature, and p–d hybridization.

We calculated the electron charge density, to describe the chemical bonding associated with the SMTO and SFTO DPs and charge sharing between the atoms, using the GGA + *U* approximation. The charge density plots describe the charge distribution difference between the optimized and initial structures. They show the loss or gain of the electron on a specific atom, blue (−0.01) and red (+0.01) in the color contour, respectively (Fig. 4). The maximum density is located in the oxygen atoms' vicinity, due to their large electronegative nature. It can also be observed that Ta atoms lose more electrons compared to the Mn/Fe atoms, agreeing with the assigned +5 and +3 valence states, respectively. The shared electrons between oxygen and Mn/Fe and Ta atoms show the covalent nature of the Mn/Fe–O and Ta–O bonds. Furthermore, the overlapping Mn, Fe, Ta and O charge contours evidence the p–d hybridization. The non-spherical shapes of the transition metal atoms Mn/Fe and Ta indicate that the d states are partially filled. Moreover, Fig. 4 visualizes the distorted and tilted Mn/FeO_6_ and TaO_6_ octahedra.

### 3.5 Optical properties

Revealing the optical properties of materials is essential to predict their possible application and device performance in optoelectronics and PVs. The optical response of matter can be described by the frequency-dependent complex dielectric function, visualizing the optical transition between the occupied and unoccupied orbitals (see Section 2 for details).

**Fig. 5:**
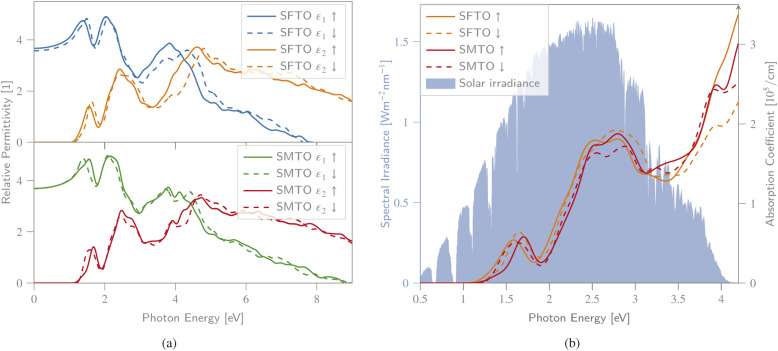
(a) Spin-dependent real *ε*_1_ and imaginary *ε*_2_ parts of dielectric function, (b) absorption spectra, as a function of photon energy calculated for SMTO and SFTO systems using GGA + *U*, together with the AM1.5g solar irradiance spectrum.^48^ All spin states exhibit a non-vanishing absorption behaviour within a wide, PV-relevant spectral range between 1.3 and 4.2 eV.

To obtain a deeper insight into the electronic band profile, and to analyse the optical behaviour, including the dielectric functions and absorption coefficients, we calculated the spin-polarized optical parameters for the SMTO and SFTO DPs, using the GGA + *U* method (*U*_Mn,Fe_ = 4.0 eV and *U*_Ta_ = 1.0 eV). Due to the studied compounds' asymmetric geometrical structure, we calculated the optical properties along *x*-, *y*- and *z*-directions. Our results, however, revealed identical values in different directions, not revealing any significant anisotropy. Hence, here we only represent the optical properties along the *x*-axis. The calculated spin-dependent real and imaginary dielectric constants, functions of energy in the energy range of 0.0 eV to 8.0 eV above the Fermi level, are plotted in Fig. 5(a). A material behaves as a dielectric for *ε*_1_ > 0 and as a metal for *ε*_1_ < 0. In Fig. 5, SMTO represents a similar dielectric behavior for the spin up and down channels due to its AFM nature. The value of the real dielectric function at zero energy shows the static dielectric constant *ε*_1_(0) that explains the reverse relation between the semiconductor band gap as *ε*_1_(0) = 1 + (*ℏω*/*E*_g_)^2^ (ref. 49). Therefore, the higher value of *ε*_1_(0) reveals a smaller band gap. For SMTO, the spin up channel has a slightly higher value of *ε*_1_(0) than the spin down channel indicating a lower band gap value as reported in Table 3. The imaginary dielectric constant is small (nearly zero) for both compounds up to certain energy. The peaks in the imaginary part of the dielectric function reveal the interband transition from the valence bands to the respective conduction bands. In SMTO and SFTO systems, the imaginary dielectric function has three main peaks at around 1.5, 2.5, and 4.5 eV. The low energy peaks are associated with the transition between the O-2p valence and the Ta-5d conduction bands. While the transition between O-2p valence band and Mn/Fe-3d conduction band caused the peaks located in higher energies. The first peak in *ε*_2_ of both compounds at 1.5 eV is attributed to the transitions from O-2p in the energy range of −1.5 to −0.5 eV to the first small peak of Ta-5d exactly above the Fermi level in the range 0.5 to 1 eV. The second peak at energy 2.5 eV in the *ε*_2_ diagram, which is stronger than the first one, is ascribed to the transitions from O-2p in the energy range of −1.5 to −0.5 eV to a strong Ta-5d peak in the energy range of 1.0 to 2.0 eV in the DOS. A significant difference between the two materials is the presence of additional Fe-3d states in the spin down channel of SFTO in the energy range between −2.0 and −1.0 eV, causing the additional electron transitions to O-2p states above the Fermi level. It gives rise to a stronger contribution of the spin down channel in the *ε*_2_ curve compared to the spin up channel at the energy ∼3.0 eV in SFTO. To investigate the visible-light harvesting capacity, we calculated the DPs' absorption coefficients, and display them alongside the AM1.5g solar irradiance reference spectrum^48^ (Fig. 5(b)). The absorption coefficient is zero when the energy is in the range of 0.0–1.0 eV, and there is no electric transition because the photon energy is below the SMTO and SFTO band gaps (∼1.0 eV). For a photon energy larger than the band gap value (∼1.0 eV), the absorption coefficient increases, reaching the first peak with the value of around 0.5 × 10^5^ cm^−1^ at 1.8 eV. The secondary peak appears at the 2.5–3.0 eV energy range, with an absorption coefficient value of 2.0 × 10^5^ cm^−1^. The absorption spectrum increases with increasing photon energy, after around 4.0 eV. The *α* value in the visible region is in the order of 10^5^ cm^−1^, confirming that SMTO and SFTO are promising candidates for PV applications.^50,51^

## 4 Conclusions

We performed DFT calculations to study the crystal structure, electronic, magnetic, and optical properties of SMTO and SFTO DPs. Our structural optimizations, using total energy calculations under the GGA-PBE scheme, indicate triclinic crystal structures for both compounds, with significant polar distortions of BO_6_ and B′O_6_ originating from the JT effect. Negative formation energies furthermore assure their thermodynamic stability. We studied the magnetic, electronic, and optical properties with the GGA + *U* method. The magnetic phase stability of SMTO and SFTO in different magnetic configurations has also been discussed, and an AFM and FM nature was predicted to be their magnetic ground states, respectively. We showed that these materials have a semiconductor behavior with an energy gap of around 1 eV according to the density of state calculations. The optical properties, including the dielectric functions and absorption coefficients, were studied. The structural stability, suitable band gap, and absorption coefficient values of studied compounds suggest they could be good candidates for PV applications.

## Conflicts of interest

There are no conflicts to declare.

## Supplementary Material
